# Stabilization of Human Multidrug Resistance Protein 4 (MRP4/ABCC4) Using Novel Solubilization Agents

**DOI:** 10.1177/2472555219867074

**Published:** 2019-08-05

**Authors:** David Hardy, Roslyn M. Bill, Alice J. Rothnie, Anass Jawhari

**Affiliations:** 1 CALIXAR, Lyon, France; 2 Life & Health Sciences, Aston University, Aston Triangle, Birmingham, UK

**Keywords:** membrane protein, stabilization, MRP4/ABCC4, calixarene detergent, SMA

## Abstract

Membrane proteins (MPs) are important drug discovery targets for a wide range of diseases. However, elucidating the structure and function of native MP is notoriously challenging as their original structure has to be maintained once removed from the lipid bilayer. Conventionally, detergents have been used to solubilize MP with varying degrees of success concerning MP stability. To try to address this, new, more stabilizing agents have been developed, such as calixarene-based detergents and styrene–maleic acid (SMA) copolymer. Calixarene-based detergents exhibit enhanced solubilizing and stabilizing properties compared with conventional detergents, whereas SMA is able to extract MPs with their surrounding lipids, forming a nanodisc structure. Here we report a comparative study using classical detergents, calixarene-based detergents, and SMA to assess the solubilization and stabilization of the human ABC transporter MRP4 (multidrug resistance protein 4/ABCC4). We show that both SMA and calixarene-based detergents have a higher solubility efficiency (at least 80%) than conventional detergents, and show striking overstabilization features of MRP4 (up to 70 °C) with at least 30 °C stability improvement in comparison with the best conventional detergents. These solubilizing agents were successfully used to purify aggregate-free, homogenous and stable MRP4, with sevenfold higher yield for C4C7 calixarene detergent in comparison with SMA. This work paves the way to MRP4 structural and functional investigations and illustrates once more the high value of using calixarene-based detergent or SMA as versatile and efficient tools to study MP, and eventually enable drug discovery of challenging and highly druggable targets.

## Introduction

Membrane proteins are capable of controlling what enters and exits cells through transporters and channels, modulating cell signaling with receptors, catalyzing reactions by utilizing enzymes, and maintaining structure with anchoring proteins. Membrane proteins are highly valuable pharmaceutical targets, and understanding the structure and function of these important proteins is vital in the production of beneficial pharmaceutical drugs. Observing membrane proteins in their native state is the most useful for producing effective pharmaceutical drugs, as there have been no modifications to the native structure. Unfortunately, membrane proteins are not stable outside of the native lipid environment. In order to gain a true understanding of how membrane proteins are structured and function, they must be viewed in isolation (purified). This means producing a highly stable membrane protein that retains its native structure through purification processes and is able to be used in functional and structural studies. To overcome this stability problem, membrane proteins have often been altered by either changing the native amino acid sequence through protein mutagenesis or engineering.^1^ Antibodies have also been used in efforts to stabilize membrane proteins,^2^ but all these methods come with a price; they might alter the native conformation of the membrane protein. Investigating membrane proteins in their native state is challenging, as they often become highly unstable when solubilized. Solubilization reagents are available in a range of strengths, from harsh anionic detergents such as sodium dodecyl sulfate (SDS) and sarkosyl, to mild zwitterionic detergents such as Fos-cholines (FC) and CHAPS, to weaker nonionic detergents such as dodecylmaltoside (DDM) and octyl glucoside (OG). All these contain a similar structure with a hydrophobic acyl tail and a hydrophilic head group, allowing them to act as amphiphiles removing the lipids surrounding the membrane protein. In doing so, they are removing the lateral pressure exerted by the lipids, keeping the membrane protein in its correct conformation and replacing it with much less stable detergents. Although these conventional detergents have been used for membrane protein studies, the success rate is variable and is largely protein dependent. Novel detergents have therefore been produced in an effort to stabilize a much greater variety of membrane proteins.

Many of these novel detergents are built on previous conventional detergents utilizing their solubilization capabilities but also enhancing their stabilizing properties. Maltose-neopentyl glycol (MNG) and glucose-neopentyl glycol (GNG) are two novel detergents that have very similar structures to DDM and OG, respectively; through the addition of a central quaternary carbon atom derived from neopentyl glycol, two hydrophobic and hydrophilic groups can be connected.^3^ Facial amphiphiles represent a slightly different approach to maintaining membrane protein stability. These molecules comprise a hydrophobic sterol backbone capable of solubilization attached to different head groups, many of which are maltose based.^3^ Facial amphiphiles have been shown to solubilize and aid in the crystallization of membrane proteins^4^ and have further been modified to create tandem facial amphiphiles and are able to span the width of a lipid bilayer.^3^

Calixarene-based detergents have been shown to have a greater ability to stabilize membrane proteins.^5,6^ They contain a calixarene platform comprising four aromatic rings, three in the *para* position and the fourth attached to the hydrocarbon chain.^6^ By altering the length of the hydrocarbon tail, the solubilization properties are affected, increasing their efficiency. Different head groups such as carboxylate or sulfonate groups can be attached to the calixarene platform. These head groups can interact, through the formation of salt bridges, with the aromatic or charged residues at the lipid–protein interface, creating a more stable membrane protein–detergent complex.^5^ A bacterial ATP-binding cassette (ABC) transporter, BmrA has previously been extracted with C4C7 (calixarene containing a seven-carbon-length hydrocarbon chain), and it maintained 90% function, whereas solubilization with DDM or FC12 resulted in a 90%–99% loss of function. Calixarenes enhance the stability of solubilized membrane proteins.^5,6^

**Figure 1.**
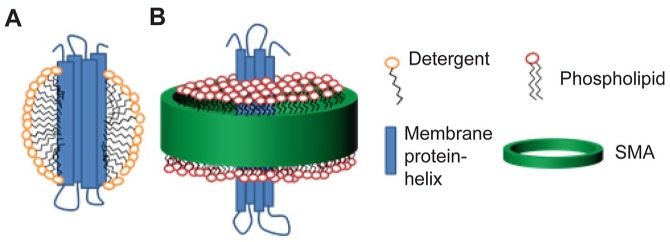
Schematic representation of membrane proteins in detergent (**A**) or in SMA (**B**). Membrane protein helices are represented in blue. SMA, phospholipids, and detergent are also indicated.

A different approach to solubilizing and stabilizing membrane proteins is through the use of a styrene–maleic acid (SMA) copolymer. This polymer is composed of alternating units of styrene and maleic acid in varying ratios. They are able to insert into membranes and solubilize bilayers, forming SMA lipid particles (SMALPs).^7,8^ Membrane proteins can become trapped inside these SMALPs, solubilizing the membrane protein and encapsulating it in a disc of its native lipids (**Fig. 1B**).^8,9^ This is very different from detergent solubilization (**Fig. 1A**). Once solubilized in SMA, the membrane protein can be purified without the need for detergents in any of the buffers, making it much more cost-effective.^10,11^ These SMALPs can also be used in functional studies as both sides of membrane proteins are accessible and have been shown to be highly stable.^10,12^ SMA has successfully been used to extract a variety of ABC transporters from different expression systems,^10,13^ along with ligand binding studies of ABC transporters and G-protein-coupled receptors (GPCRs),^12^ functional reconstitution of KscA,^11^ and lipid analysis. These examples show the versatility of the SMA polymer in aiding functional understanding of membrane proteins. SMALP-purified proteins have been used for structural studies by both crystallography^14^ and electron microscopy.^10,15–17^

Most reported membrane protein structure and function studies describe the use of one main reagent. Therefore, a comparative study is very often missing. In the current work, we have applied classical detergents, calixarene-based detergents, and SMA polymers to investigate in parallel the solubilization and stabilization of the same membrane protein target. We have chosen a member of the ABC transporter family (ABCC4/MRP4) as a model membrane protein. ABC transporters are integral membrane proteins that are found in all types of organisms, from prokaryotes to humans. They utilize energy from ATP binding and hydrolysis to transport a variety of substrates across the biological lipid bilayer.^18^ MRP4 is made of four core domains: two transmembrane domains (TMDs) and two nucleotide binding domains (NBDs). Each of the two TMDs comprise six transmembrane alpha helices, and the two NBDs have binding sites for ATP that are homologous throughout the superfamily, unlike the TMDs.^19^ As its name suggests, MRP4 can confer resistance to drugs, including cancer chemotherapy, antivirals, and antibiotics.^20^ It can also transport signaling molecules such as cyclic nucleotides and eicosanoids, making it a drug target for inflammation, pain,^21^ and cardiovascular disease,^22^ and it has also been implicated in the development of cancer.^23,24^ To date, there is no known structure of human MRP4. To investigate MRP4 structure and function, it is critical to express it, solubilize it, and purify it while maintaining its functionality and structural integrity. Here we show that using calixarene-based detergent or SMA allowed the purification of very stable, homogenous MRP4.

## Materials and Methods

### Expression and Membrane Preparation

Expression of the recombinant human MRP4-his_6_ within *Sf*9 cells was conducted using a baculovirus encoding for recombinant MRP4 generated from a pFastBac-MRP4-his_6_ construct with a C-terminal hexa-his-tag. Typically, *Sf*9 insect cells were expressed using the Bac-to-Bac Baculovirus System (ThermoFisher, Waltham, MA) for 48 h at a multiplicity of infection (MOI) of 2. Infected *Sf*9 cells were harvested by centrifugation at 6000*g* for 10 min. The cell pellet was resuspended in homogenization buffer (50 mM Tris-HCl, pH 7.4, 250 mM sucrose, 0.25 mM CaCl_2_) and protease inhibitors (1.3 µM benzamidine, 1.8 µM leupeptin, 1 µM pepstatin). Homogenization of S*f*9 cells was carried out using nitrogen cavitation at 500 psi for 15 min on ice. The cell lysate was centrifuged at 750*g* for 10 min; the supernatant was then subsequently centrifuged at 100,000*g* for 20 min. The membrane pellet was resuspended in TSB buffer (50 mM Tris-HCl, pH 7.4, 250 mM sucrose) and stored at −80 °C.

### Solubilization

SMA polymers were obtained from Cray Valley (SMA 2000, Exton, PA) or Polyscope (SZ25010, Geleen, Netherlands) as styrene–maleic anhydride polymers and were hydrolyzed in 1 M NaOH, refluxed, and freeze-dried to form the SMA form as described in Rothnie.^13^

Dot blot analysis was carried out by solubilizing MRP4 *Sf*9 membranes at 5 mg/mL total protein using 10× critical micellar concentration (CMC) of each detergent (calixarene-based detergents, CALIXAR (Lyon, France), or conventional detergents; VWR, Lutterworth, UK) or 2.5% (w/v) for SMA polymers, for 2 h at 4 °C. Two hundred microliters was loaded into the dot blot apparatus (Bio-Rad, Watford, UK), filtered through nitrocellulose, and washed 3× with 200 µL of phosphate-buffered saline (PBS) to remove insoluble material. An anti-his horseradish peroxidase (HRP) antibody (3:2000; R&D Systems, Abingdon, UK) was used to detect MRP4 and was quantified on a ChemiDoc imaging system. Solubility efficiency was calculated by comparing the density of the dot to an SDS control. Western blot analysis was performed to assess the solubilization efficiency. *Sf*9 MRP4 membranes at 5 mg/mL total protein were solubilized using 10× CMC detergent or 2.5% (w/v) SMA polymer at 4 °C for 2 h and then centrifuged at 100,000 g. The insoluble pellet was resuspended in 1% (w/v) SDS. Samples were run on SDS-PAGE (polyacrylamide gel electrophoresis) and transferred to a polyvinylidene fluoride (PVDF) membrane, and a primary anti-his antibody was used to detect MRP4. Solubility efficiency was calculated via densitometry analysis using ImageJ or Image Studio analysis software by calculating the intensity of the solubilized band as a percentage of the total (soluble + insoluble). Optical density (OD) readings were measured on an Ultrospec 2000 spectrophotometer (Pharmacia Biotech) at 600 nm.

### Western Blot-Based Thermal Shift Assay

The thermostability of solubilized or purified MRP4 was measured by heating samples for 10 min at different temperatures (4–90 °C) and then centrifuging them at 14,000*g* for 10 min. The supernatant was removed and analyzed by Western blot. Data fitting was performed by fitting a dose (temperature) versus normalized response curve using GraphPad Prism. The method was previously described and applied to GPCR solubilization.^25^

### MRP4 Purification

*Sf*9 MRP4 membranes solubilized with 10× CMC calixarenes were mixed with Ni-NTA affinity resin at a volumetric ratio of 10:1 (soluble MRP4/resin) for 2 h at 4 °C. Resin was washed with 3 × 10 column volumes (CV) of 5 mM imidazole and eluted in 5 × 1 CV using 200 mM imidazole. All purification buffers were supplemented with two CMC CALIXAR detergents. MRP4 was concentrated using a 50 kDa MWCO spin concentrator.

Affinity purification using an SMA polymer was performed using an adapted protocol from Rothnie.^13^ *Sf*9 MRP4 membranes solubilized with 2.5% SMA 2000 were mixed with Ni-NTA resin overnight at 4 °C at a volumetric ratio of 20:1 (soluble MRP4/resin). Resin was washed with 5 × 10 CV of 20 mM imidazole and eluted in 5 × 1 CV using 200 mM imidazole. MRP4 was concentrated using a 30 MWCO spin column.

## Results

### MRP4 Solubilization

**Figure 2.**
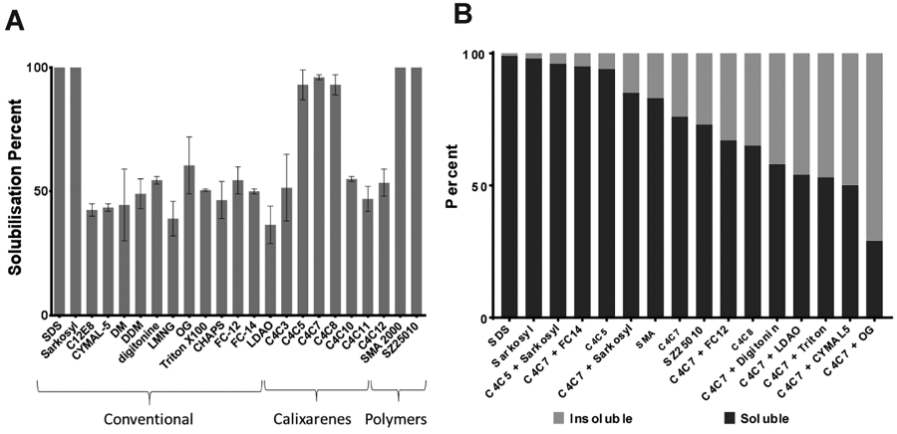
Solubilization of MRP4. (**A**) Screening of conventional detergents, calixarenes, and SMA polymers for MRP4 solubilization analyzed by dot blot using a his-tag antibody. All dot intensities were compared with an SDS control. Data are the mean and variation between duplicates. (**B**) Solubilization efficiency assessed by Western blot for the selected conditions from dot blot analysis.

We wanted to find out which detergents or polymers were best for both solubilizing and stabilizing MRP4. We started by measuring their solubilization properties by screening a large range of detergents and SMA polymers monitored by dot blot (**Fig. 2A**). All conventional detergents screened were able to solubilize around 50% of MRP4 from *Sf*9 cell membranes with the exception of harsh anionic sarkosyl, which was equal to SDS. The calixarene detergents tested here had the same calix[4]arene platform onto which three acidic methylene–carboxylate groups had been grafted at the *para* position, while the other face bears a single aliphatic chain of different lengths. Typically, C4C5, C4C7, and C4C8 correspond to detergents with five-, seven-, and eight-carbon lengths, respectively. C4C5, C4C7, and C4C8 were able to solubilize more than 90% of the MRP4. Anything below (C4C3) or above (C4C10, C4C11, and C4C12) decreased the solubilization efficiency by around half and was comparable to conventional detergents. The length of the acyl tail in calixarene detergents therefore plays a key role in solubilization efficiency. The SMA polymers also had high solubilization efficiency with SMA 2000 and SZ25010 both at 100%. These results show that novel solubilizing agents are capable of greatly increasing the solubilization efficiency of MRP4 when compared with conventional detergents. The top conditions with the highest solubility efficiency revealed by dot blot were chosen to measure and confirm the solubility efficiency using Western blot analysis (**Fig. 2B**). C4C5 was shown to have a very high solubility efficiency of 90%; C4C7 was at 76% and C4C8 was at 65% solubility, again showing that the length of the acyl tail can affect solubility efficiency. The SMA 2000 and SZ25010 solubility efficiencies were at 83% and 73%, respectively. Only detergent mixtures with harsh conventional detergents such as sarkosyl and FC12 allowed higher solubilization efficiency than calixarene detergents alone. Interestingly, the kinetics of MRP4 solubilization by SMA 2000 were much faster than is typically reported for SMA polymers (**Suppl. Fig. S1**),^10^ and it can be seen that the vast majority of solubilization events occur within the first 15 min.

### Thermostability of Solubilized MRP4

**Figure 3.**
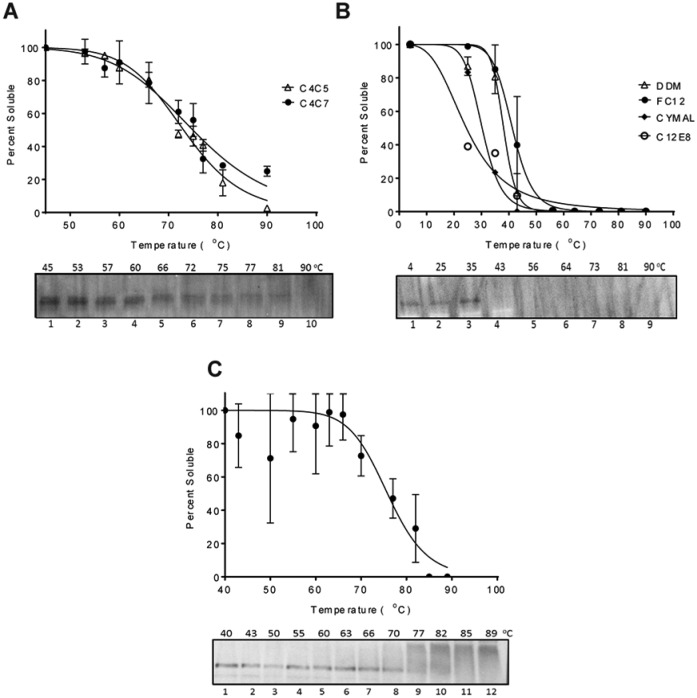
Thermostability of MRP4. Thermostability was measured by the percent of soluble MRP4 present after heating. (**A**) Thermostability of MRP4 solubilized with calixarenes C4C5 and C4C7 from 45 to 95 °C (*n* = 2). (**B**) Conventional detergent thermostability from 4 to 90 °C (*n* = 2). (**C**) Thermostability results for MRP4 solubilized with SMA 2000 from 40 to 89 °C (*n* = 2). The Tm was calculated as the temperature at which 50% remained soluble. Dose (temperature) versus normalized response curve fitted for all graphs. Data represent the mean and variation between duplicates.

Before moving on to purification, the ability of these solubilizing agents to stabilize MRP4 was assessed using a previously established Western blot-based thermal shift assay.^25^ This assay relies on the assumption that unstable heated proteins will aggregate, and after ultracentrifugation and Western blot, the band intensity corresponding to the protein will decay proportionally to its instability. By measuring the amount of MRP4 that remains soluble at increasing temperatures, the denaturing point (Tm), 50% soluble, can be estimated. MRP4 solubilized using either C4C5 or C4C7 showed a very high Tm of around 70 °C (**Fig. 3A**). SMA 2000-solubilized MRP4 also showed a very high Tm of around 75 °C (**Fig. 3C**). The conventional detergents chosen all have a similar solubilization percentage and had previously been used in studies involving the solubilization of ABC transporters. The Tm for conventional detergents ranged from 28 to 40 °C, with FC12 being the highest and C12E8 the lowest (**Fig. 3B**). Thus, the Tm for MRP4 in the calixarene detergents (C4C5 and C4C7) or SMALPs was 30 or 35 °C higher than that of the best conventional detergent for MRP4 stability, respectively.

### MRP4 Purification

**Figure 4.**
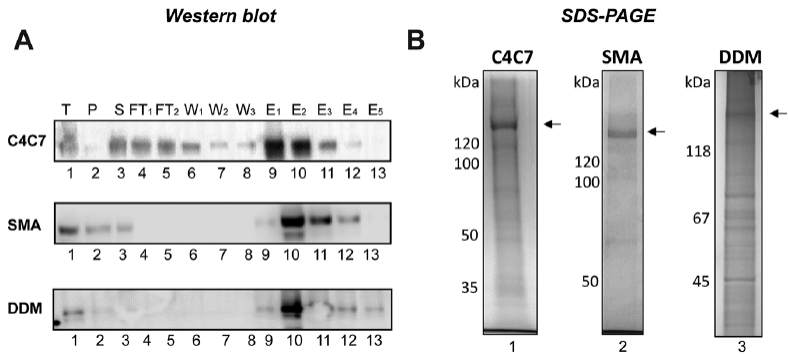
MRP4 purification. (**A**) Western blot showing his-tag affinity purification of MRP4 after solubilization using C4C7, SMA 2000, or DDM. (**B**) Instant Blue-stained SDS-PAGE of purified MRP4 using C4C7, SMA 2000, or DDM.

To evaluate the impact of solubilization reagents on protein purification, his-tag affinity purification was performed for MRP4 solubilized with either C4C7, SMA 2000, or DDM. **Figure 4A** shows that MRP4 was specifically loaded and eluted from the Ni-NTA affinity column. SDS-PAGE gels demonstrate a relatively pure MRP4 after one affinity purification step for both C4C7 and SMA 2000 (**Fig. 4B**). However, with DDM, the MRP4 is not at all pure, with multiple contaminating bands that are equally as intense as the MRP4 band (**Fig. 4B**). Notably, C4C7 consistently gave higher yields of pure protein (184 ± 8 µg/L cell culture) compared with SMA 2000 (26 ± 7 µg/L cell culture).

**Figure 5.**
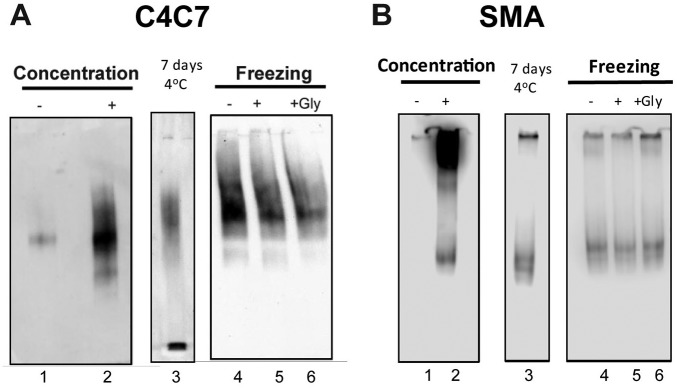
Native gel analysis. Native PAGE and Western blot analysis to assess the behavior in solution of MRP4 purified with C4C7 (**A**) or SMA 2000 (**B**). It was examined after protein concentration using centrifugal filter concentrators and stored at 4 °C for 7 days or after freezing/thawing steps. +Gly corresponds to the addition to 10% glycerol before the freezing and thawing steps.

Native PAGE Western blot analysis (**Fig. 5**) confirmed that one main nonaggregated population of MRP4 was obtained for both C4C7 and SMA 2000, demonstrating the homogeneity and good behavior in solution when SMA 2000 or C4C7 was used for solubilization and purification. MRP4 solubilized and purified in C4C7 could easily be concentrated using a centriprep centrifugal filter unit without generating any aggregates since the MRP4 band becomes more intense upon concentration, with no aggregate species observed on the well of the gel (**Fig. 5A**, compare lane 2 with lane 1). For both C4C7 and SMA 2000, storage for 7 days at 4 °C had no effect on MRP4 aggregation. Similarly, MRP4 showed no changes after freezing and thawing steps in C4C7 or SMA 2000. The same aggregate-free behavior in solution was observed even in the absence of cryoprotectant (10% glycerol) (**Fig. 5**).

**Figure 6.**
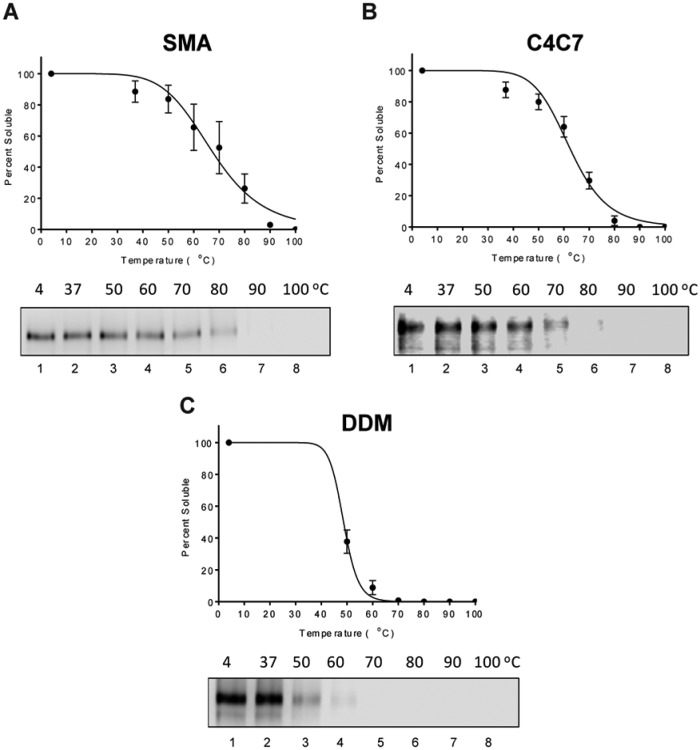
Thermostability of purified MRP4. Thermostability curves of MRP4 purified in SMA (**A**), C4C7 (**B**), and DDM (**C**), respectively, based on percent soluble after heating and centrifugation (*n* = 3 ± SEM). The blue line represents the transition/melting point (Tm); the temperature at 50% of MRP4 is still soluble, as previously described.^25^ The Western blots below graphs **A**, **B**, and **C** show examples of the amounts of soluble MRP4 at each temperature in SMA, C4C7, and DDM, respectively. The density of each band was taken and compared with the 4 °C control to calculate the percent soluble.

The thermostability of purified MRP4 was also examined using the same Western blot thermal shift assay as previously described. Interestingly, the same tendencies were noticed after solubilization and purification of MRP4 (**Fig. 6**). In fact, SMA 2000 and C4C7 maintained high stability of MRP4 during purification, and the Tm remained high at 71 and 65 °C, respectively (**Fig. 6A,B**), whereas MRP4 solubilized and purified in DDM has a Tm of 49 °C (**Fig. 6C**).

## Discussion

Here we show that MRP4 was successfully solubilized and purified using both C4C7 and SMA 2000. Concerning calixarene-based detergent, the length of the hydrophobic chain controls solubilization efficiency. MRP4 was kept in a very stable state compared with the best of the conventional detergents, DDM or FC12. Indeed, the stabilizing properties of C4C7 and SMA 2000 were demonstrated by a dramatic thermostability improvement of 30 and 35 °C, respectively, at the solubilization step in comparison with the best conventional detergents. This stabilization shift is very high considering that the MRP4 investigated was a full-length wild-type without any single point mutation. It is very common to heavily mutate membrane proteins to improve their thermostability.^26^ This was the case for the adenosine receptor, which was mutated at eight residues and had 96 amino acids at the carboxy-terminal deletion, leading to ~15 °C stability improvement.^27^ Our results illustrate the fact that there is no need to systematically mutate or truncate membrane proteins in order to stabilize them. Using favorable chemical environments can positively impact the stability and functionality of membrane proteins. The fact that C4C7 detergent exhibits comparable overstabilizing features in comparison with SMA is outstanding considering that in contrast to detergents, SMALPs contain lipids, and it is well accepted that lipids exert a stabilizing effect on membrane proteins.^28^ The fact that stability improvement was reduced (to +16 and +22 °C for C4C7 and SMA, respectively, in comparison with DDM) when MRP4 was solubilized and purified, in comparison with solubilized only, may be due to the loss of some key stabilizing lipids during the purification process. Further studies including mass spectrometry analysis are required to confirm that. Differences in the degree of cooperativity of the thermal shift curves were noticed. This was also observed for other membrane proteins using other detergents.^29^ Further studies using other thermostability assays are required to explain the contribution of the chemical environment (lipid/detergent/polymer) and the membrane protein dynamics (in detergent or in SMA) on the shape of thermal shift curves.

Now that good solubilization and stabilization conditions have been found, structural investigation of MRP4 can begin. The next steps would be to use cryo-EM to investigate MRP4 structure in solution. Preliminary negative stain electron microscopy images have shown isolated particles of MRP4 (data not shown). SMA and calixarene-based detergent have both previously been shown to be compatible with electron microscopy.^15–17,30^ It has previously been reported that SMA somehow interferes with binding to Ni^2+^ resin,^13,31,32^ and this might explain the difference in protein yield. It has also been previously reported that SMA 2000 results in much more pure protein samples than conventional detergents.^10,33^ Here it is shown that SMA 2000 gave a good degree of purity following a single-step affinity purification, but, in contrast to more conventional detergents, C4C7 also gave a comparable degree of purity. There were, however, some limitations found for each approach. Due to its calixarene platform, calixarene detergent absorbs at 280 nm, which makes protein quantification difficult and limits the use of some biophysical charaterization, such as circular dichroism or tryptophan fluoresence. To adress this limitation, new classes of compounds with similar architecture but without the calixarene platform have been designed and applied successfully to membrane protein stabilization,^34^ and this could be a promising future direction for MRP4 studies. The addition of mild groups such as saccharide heads or cholesterol-like groups provides more diversity for such classes of stabilizing detergents.^6,35^ Current limitations to the SMA approach include the disc size. The typical size is 10–12 nm in diameter, which may mean some large proteins or protein complexes will not fit. However, recent reports have suggested that there is some flexibility with this.^16^ SMA is also sensitive to divalent cations such as magnesium and calcium.^33^ This is particularly problematic for proteins like the ABC transporters, which require Mg^2+^ for ATP hydrolysis. Alternative polymers such as styrene maleimide (SMI) and diisobutylene–maleic acid (DIBMA) have also being developed that are reported to overcome the divalent cation sensitivity by either replacing the maleic acid with maleimide (SMI) or replacing the styrene with diisobutylene (DIBMA).^36,37^

Taken together, we report here that if used for solubilization and purification, C4C7 and SMA maintain the structural integrity of MRP4. In addition to structural studies, these findings open up the possibility of functional and drug discovery approaches with MRP4. Calixarene-based detergents and SMA represent important versatile tools as part of the fast-growing toolbox to help unlock the drug discovery potential of challenging membrane protein targets. This is and will undoubtedly be the case for antibody discovery, structure-based drug design, and small-molecule screening of highly druggable membrane proteins

## Supplemental Material

DS_DISC867074 – Supplemental material for Stabilization of Human Multidrug Resistance Protein 4 (MRP4/ABCC4) Using Novel Solubilization Agents Click here for additional data file. Supplemental material, DS_DISC867074 for Stabilization of Human Multidrug Resistance Protein 4 (MRP4/ABCC4) Using Novel Solubilization Agents by David Hardy, Roslyn Bill, Alice Rothnie and Anass Jawhari in SLAS Discovery
