# Supplementary material for: Stabilization of Human Multidrug Resistance Protein 4 (MRP4/ABCC4) Using Novel Solubilization Agents
Source: SLAS Discov. 2019 Aug 5;24(10):1009–17. doi: 10.1177/2472555219867074 (PMC6873219; doi:10.1177/2472555219867074)
Supplement: DS_DISC867074 – Supplemental material for Stabilization of Human Multidrug Resistance Protein 4 (MRP4/ABCC4) Using Novel Solubilization Agents [file DS_DISC867074.pdf]

Stabilization of human multidrug resistance protein 4 (MRP4/ABCC4) using novel solubilization agents.

David Hardy, Roslyn. M.Bill, Alice. J. Rothnie & Anass Jawhari

Supplementary Information

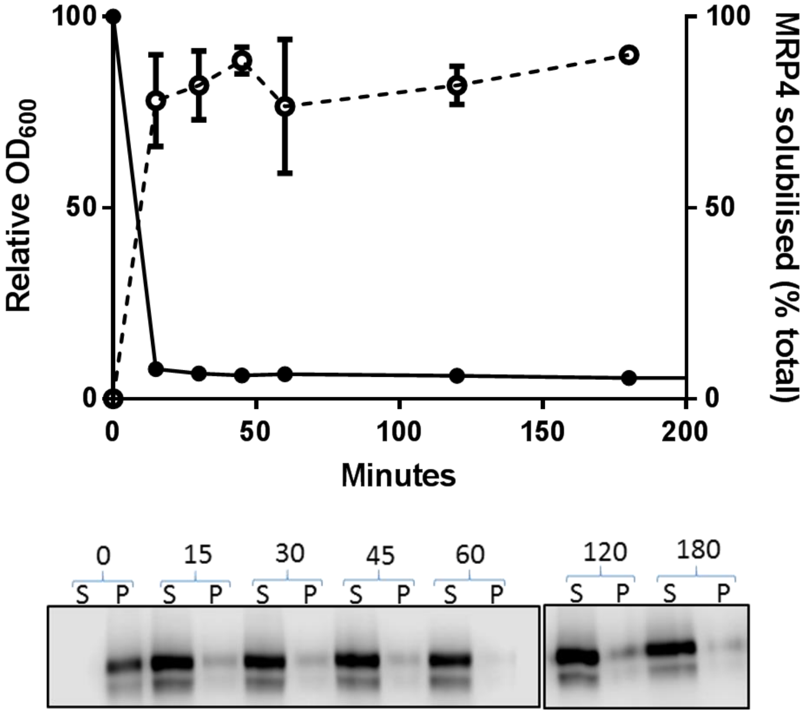

Supplementary Figure 1: Kinetics of MRP4 solubilisation with SMA 2000. The solubilisation of MRP4 expressing *Sf*9 cell membranes at room temperature was monitored over time by measuring both the turbidity/OD_{600} (closed circles) to monitor general membrane disruption, and Western blotting to measure MRP4-specific solubilisation (open circles). It can be seen that the solubilisation was quick, and essentially complete after 15 minutes.
